# Global transcriptomic response of *Escherichia coli* to *p*-coumaric acid

**DOI:** 10.1186/s12934-022-01874-6

**Published:** 2022-07-20

**Authors:** José Ignacio Rodríguez-Ochoa, Juan Carlos Fragoso-Jiménez, Noemí Flores, Luz María Martínez, Francisco Bolivar, Alfredo Martinez, Guillermo Gosset

**Affiliations:** Departamento de Ingeniería Celular y Biocatálisis, Instituto de Biotecnología, Universidad Nacional Autónoma de México, Cuernavaca, Morelos México

**Keywords:** Transcriptome, *p*-Coumaric acid, Aromatic compounds, Toxic compounds, Efflux systems

## Abstract

The aromatic compound *p*-coumaric acid (p-CA) is a secondary metabolite produced by plants. This aromatic acid and derived compounds have positive effects on human health, so there is interest in producing them in biotechnological processes with recombinant *Escherichia coli* strains. To determine the physiologic response of *E. coli* W3110 to p-CA, dynamic expression analysis of selected genes fused to a fluorescent protein reporter as well as RNA-seq and RT-qPCR were performed. The observed transcriptional profile revealed the induction of genes involved in functions related to p-CA active export, synthesis of cell wall and membrane components, synthesis of amino acids, detoxification of formaldehyde, phosphate limitation, acid stress, protein folding and degradation. Downregulation of genes encoding proteins involved in energy production, carbohydrate import and metabolism, as well as several outer and plasma membrane proteins was detected. This response is indicative of cell envelope damage causing the leakage of intracellular components including amino acids and phosphate-containing compounds. The cellular functions responding to p-CA that were identified in this study will help in defining targets for production strains improvement.

**Supplementary Information:**

The online version contains supplementary material available at 10.1186/s12934-022-01874-6.

## Introduction

The phenylpropanoids are a large family of compounds synthesized by plants, having roles mainly related to protection from biotic and abiotic stress and as precursors of lignin [1]. The shikimate pathway provides the aromatic amino acids phenylamine and tyrosine, the precursors of the phenylpropanoid pathway. The enzymes phenylalanine ammonia lyase (PAL) and tyrosine ammonia lyase (TAL) catalyze the non-oxidative deamination of their substrates to generate *trans*-cinnamic acid and *p*-coumaric acid (p-CA), respectively. In plants, *trans*-cinnamic acid is the main precursor of the phenylpropanoid pathway. The enzyme cinnamate 4-hydroxylase (C4H) transforms *trans*-cinnamic acid to p-CA, the branchpoint in phenylpropanoid biosynthesis [2].

The aromatic acid p-CA is a dietary micronutrient. It is a valuable compound having anti-inflammatory and antioxidant properties [3, 4]. More recently, anxiolytic, antiproliferative, neuroprotective and nephroprotective effects have been reported for this compound [5–8]. This aromatic acid is a precursor of chemicals used for the synthesis of novel adhesives, coatings, thermoplastics, aromas, flavoring agents, cosmetics, nutrition, and health products [9, 10]. It is known that p-CA can be found in most plant species. However, extraction from plant sources is a complex process, mostly because this compound is found at a low concentration. A viable alternative to overcome the limitations of traditional extraction methods is based on the generation of engineered microorganisms [11]. The microbial species routinely employed in biotechnology lack the natural capacity to synthesize phenylpropanoids. To overcome this limitation, genetic engineering has been employed to generate microbial p-CA production strains. The basic strategy for generating these strains is based on modifying the native cell metabolism to increase flow into the tyrosine or phenylalanine biosynthetic pathways and expressing heterologous genes encoding enzymes with PAL, TAL, and 4-hydroxylase activities. These efforts have concentrated on microbial species including *Escherichia coli*, *Pseudomonas putida*, *Streptomyces lividans* and *Saccharomyces cerevisiae*. Engineered strains have been generated to produce p-CA from simple carbon sources at concentrations ranging from 2.3 to 1740 mg/L [12]. It is expected that improvements regarding strain engineering and production process should result in important enhancements in the yields and titers for p-CA. However, this aromatic acid is a toxic compound. Therefore, its accumulation in the culture broth under production conditions can have a negative effect on cell viability and productivity. For *E. coli*, it has been determined that growth is completely arrested at a p-CA concentration of 10 g/L [10].

Microorganisms possess diverse mechanisms to cope with toxic compounds. In *E. coli*, protein TolC is an outer membrane channel that is required for the function of several efflux systems that are involved in the export of toxic compounds [13]. Protein TolC is necessary for the function of AcrAB, the main multidrug efflux system in *E. coli* that exports several types of toxic compounds [14]. A mutant in *acrAB* displayed increased sensitivity to p-CA, with a minimal inhibitory concentration (MIC) of 25 mM, showing that this compound is a substrate of the efflux system. When *tolC* was deleted, the MIC was 6 mM, this result suggests that there are other efflux systems for p-CA that employ the TolC channel [10]. In another study with *E. coli*, transcriptome analysis in the presence of *p*-hydroxybenzoic acid (pHBA) found upregulation of genes *aaeA* and *aaeB* that are part of operon *aaeXAB*. Compounds pHBA, 1-naphthoate, benzoate and salicylate also induced expression of this operon. In a study to find genes responsive to lignin-hydrolysate compounds, operon *aaeXAB* was induced in the presence of ferulic acid, ferulaldehyde, vanillic acid and p-CA [15]. Genes *aaeXAB* encode an efflux system that can employ as substrates the hydroxylated aromatic carboxylic acids pHBA, 6-hydroxy-2-naphthoic acid, p-CA, 1,5-dihydroxynaphthalene, 1,6-dihydroxynaphthalene, 2,7-dihydroxynaphthalene, 2-naphthoic acid and *trans*-cinnamic acid [16]. It was determined that plasmid overexpression of operon *aaeXAB* resulted in a two-fold increase in resistance to p-CA [10]. Gene *aaeR* is adjacent and transcribed divergently from operon *aaeXAB*, it encodes a regulatory protein from the LysR family. It was determined that *aaeR* is required to observe induction of *aaeXAB* by pHBA [16]. Gene *aaeR* has also been designated as *qseA* and it has been found to participate in quorum sensing [17, 18].

The genome-wide identification in *P. putida* of tolerance mechanisms to p-CA has been determined. In this study, *P. putida* was found to be more tolerant to p-CA when compared to *E. coli*. Genes including the ABC transporter Ttg2ABC, the cytochrome c maturation system and genes involved in membrane stability were identified to play an important role in p-CA tolerance [19].

The presence of aromatic acid compounds, both in the natural environment and in biotechnological production processes, causes a physiological response in microorganisms. Understanding how an organism copes with an aromatic toxic compound can help in designing strategies for production strain improvement. It is also important to understand how microorganisms in the human gut tolerate and chemically transform aromatic compounds from the human diet. In this regard, studies based on determining the global transcriptional response to aromatic compounds are providing useful information. For p-CA, there is currently only one global transcriptome report. The model probiotic organism *Lactobacillus plantarum* is a human lactic acid bacterium that can be found in the colon. To provide further knowledge on the physiological responses to p-CA, transcriptional profiling was employed with a human isolate of *L. plantarum* [20]. Such study revealed the induction of genes encoding functions related to stress resistance and detoxification, including chaperones, proteases, multidrug transporters, heat shock and alkaline shock proteins. Downregulation of genes encoding functions related to translation, pyrimidine synthesis, carbon transport and metabolism, cell wall and membrane-associated functions was also observed. The study provided insights on the global effects of p-CA on *L. plantarum* and helped in understanding how this gut commensal bacterium might be involved in the metabolism of p-CA [20].

The bacterium *E. coli* is both a biotechnological producer of p-CA and in the human gut can be exposed to this aromatic acid. It is therefore relevant to determine the physiological response of this bacterium to the presence of p-CA, as this will help in understanding how this organism copes with this aromatic toxic compound. This knowledge has the potential to be applied for improving current microbial p-CA production strains. Also, determining how this compound alters the physiology of *E. coli* should shed light on the effect of dietary aromatic acids on bacterial gut symbionts. In this study, we performed dynamic measurements of promoter activity for selected genes as well as RNAseq to determine the global transcriptomic response of *E. coli* strain W3110 to a sub-lethal concentration of p-CA.

## Material and methods

### Strains, plasmids and cultivation conditions

The *E. coli* strains used in this work are listed in Table 1. Strain W3110 was employed in p-CA growth and gene expression analyses [21]. *E. coli* BW25113 as well as mutants lacking genes *aaeX*, *aaeA*, *aaeB*, *aaeR*, *dnaK*, *clpB*, *clpP*, *htpG*, *tolC*, *acrA*, *acrB*, *marR*, *marA* or *inaA* were obtained from the Keio collection [22]. The low copy plasmid pUA66 is the basis of a reporter library containing gene *gfpmut2*, encoding a fast-folding version of GFP, fused to upstream regions of genes from *E. coli* [23]. Derivatives of plasmid pUA66 employed in this study contained the upstream promoter regions of the following genes, *aaeR*, *clpB*, *clpP*, *dnaK*, *dnaJ*, *groE*, *htpG*, *inaA* and *marR*, identified in this library as AZ07/H6, AZ04/D12, AZ20/H10, AZ20/E8, AZ20/F8, AZ20/F9, AZ20/B9, AZ11/D10, AZ13/E2, respectively [23]. W3110 transformed with derivatives of plasmid pUA66 were supplemented with kanamycin 30 μg/mL. For growth kinetics and fluorescence measurement experiments, the strain preinoculum was cultured overnight in a tube at 300 rpm and 37 °C with 4 mL of Luria–Bertani medium. A volume was taken to inoculate at an OD_600_ of 0.1 a tube with 4 mL of minimal M9 medium composed of 6 g/L Na_2_HPO_4_, 3 g/L KH_2_PO_4_,0.5 g/L NaCl, 1 g/L NH_4_Cl, 0.5 g/L MgSO_4_, 0.01 g/L CaCl_2_, 0.01 g/L thiamine hydrochloride. The trace elements solution contained the following: 1.5 g/L Na_2_EDTA · 2H_2_0, 0.45 g/L ZnSO_4_ · 7H_2_0, 0.03 g/L MnCl_2_ · 4H_2_0, 0.1 g/L H_3_BO_3_, 0.04 g/L Na_2_MoO_4_ · 2H_2_0, 0.3 g/L FeSO_4_ · 7H_2_0, and 0.03 g/L CuSO_4_ · 5H_2_0. As carbon source, glycerol was added at a final concentration of 10 g/L. Solutions of p-CA were made either in milli Q sterile water adjusted at pH 7 with NaOH 1 M to a final p-CA concentration of 190 mM or in absolute ethanol to a final p-CA concentration of 500 mM. Growth kinetics were determined by measuring OD_600_ every 20 min for 24 h on a microtiter plate reader (Biotek, Vermont, USA). Culture volume was 150 μL on each microtiter plate well and it was inoculated at an OD_600_ of 0.05 from a culture adapted in M9 medium. Cultures were incubated at 37 °C and 567 rpm. To determine the fluorescence of strains carrying derivatives of plasmid pUA66, they were cultivated in microtiter plate wells as explained above. The fluorescence was measured by excitation at 410 nm and reading emission at 520 nm. The p-CA dissolved in ethanol was added 5 h after starting the cultures at a concentration of 1.5, 3 or 5 mM. To eliminate the autofluorescence signal, the strain carrying plasmid pUA66 without a promoter region was included in each plate and in the same conditions for each p-CA concentration tested. The fluorescence value of this control strain was subtracted from the value of strains with promoter region fusions for all read times and p-CA concentrations. For control cultures, the same amount of ethanol used for p-CA solubilization was added. All cultures were performed in triplicate.

**Table 1:** *E. coli* strains and plasmids employed in this study

Name	Description	Source
*Strains*		
W3110	*E. coli* F- λ- *rph-1 IN(rrnD-rrnE)*1	[21]
BW25113	*E. coli* F- *λ- rph-1 Δ(araD-araB)567 ΔlacZ4787(::rrnB-3) Δ(rhaD-rhaB)568 hsdR514*	[22]
BW25113ΔaaeX	Deletion of gene *aaeX*	[22]
BW25113ΔaaeA	Deletion of gene *aaeA*	[22]
BW25113ΔaaeB	Deletion of gene *aaeB*	[22]
BW25113ΔdnaK	Deletion of gene *dnaK*	[22]
BW25113 ΔclpB	Deletion of gene *clpB*	[22]
BW25113 ΔclpP	Deletion of gene *clpP*	[22]
BW25113 ΔhtpG	Deletion of gene *htpG*	[22]
BW25113ΔaaeR	Deletion of gene *aaeR*	[22]
BW25113ΔtolC	Deletion of gene *tolC*	[22]
BW25113ΔacrA	Deletion of gene *acrA*	[22]
BW25113ΔacrB	Deletion of gene *acrB*	[22]
BW25113ΔmarA	Deletion of gene *marA*	[22]
BW25113ΔmarR	Deletion of gene *marR*	[22]
BW25113ΔinaA	Deletion of gene *inaA*	[22]

### RNA-seq experiments

Cultures were carried out in triplicate with strain W3110 in 250 mL baffled shake flasks with 50 mL of M9 medium supplemented with glycerol 10 g/L at 300 rpm and 37 °C. When cultures reached an OD_600_ of 1.0, 500 μL of a solution of p-CA 500 mM in ethanol was added and samples for RNA purification were taken after 20 and 60 min. In a control experiment without the addition of p-CA, a sample was taken at 60 min after the culture reached an OD_600_ of 1.0 In another control, 500 μL of ethanol was added when the culture reached an OD_600_ of 1.0 and a sample was taken after 60 min. Samples of 10 mL of culture media were taken and mixed with 0.5 ml of RNA Later (Ambion Inc., USA). The mixture was then centrifuged for 5 min at 4 °C at 8000 rpm and the pellet was kept frozen at −70 °C until use.

### RNA purification, mRNA library construction and RNA-seq

Total RNA was extracted by following the hot phenol method [22]. Elimination of DNA was carried out using DNAse I kit (Fermentas, Burlington, Canada). RNA samples were observed in 2% agarose gel and RNA A260/A280 and A260/A230 ratios were ≥ 1.8. The RNA integrity (RNA Integrity Number; RIN) was determined by the Bioanalyzer 2100 system (Agilent Technologies, Inc., Santa Clara,CA) using an Agilent RNA 6000 Nanochip, RIN values were obtained for each RNA sample and we only considered those with a value larger than 8. Ribosomal RNA elimination and cDNA library construction was performed with Zymo-Seq RiboFree™ Total RNA Library Kit (Zymo Research, Irvine, U.S.A.) following manufacturer instructions. The transcriptome data have been deposited in the European Nucleotide Archive at EMBL-EBI under accession number PRJEB48364 (https://www.ebi.ac.uk/ena/browser/view/PRJEB48364).

### Differential expression analysis

RNA-seq reads were quantified by employing the Salmon software using default parameters with the *E. coli* K-12 substr. W3110 genome cds file as reference (ftp://ftp.ncbi.nlm.nih.gov/genomes/all/GCF/000/010/245/GCF_000010245.2_ASM1024v1/GCF_000010245.2_ASM1024v1_cds_from_genomic.fna.gz). Differential expression analysis was carried out using edgeR [24]. We used the expression level of cells growth without p-CA and sample taken at 60 min as reference for every contrast. In addition, we used Benjamini–Hochberg as the *p*-value adjust method [24]. Differentially expressed genes were which ones that had a *p*-value < 0.01, we used a twofold-change logarithm as cut-off, positive and negative. We obtained a list of differentially expressed genes for each p-CA time exposure against the control condition. Afterward, we converted the RefSeq identifiers in Uniprot database (http://www.uniprot.org) using the Retrieve/ID mapping tool to obtain genes names, descriptions, accession numbers of Uniport and bnumbers.

### Annotation of differentially expressed genes

The differentially expressed genes were annotated in the Cluster of Orthologous Groups (COG) using the EggNog-mapper (http://eggnog-mapper.embl.de/), the settings were the Gamma proteobacteria as taxonomic scope, orthology restrictions were one-to-one orthology only. We only considered the annotations with experimental evidence and a minimum e-value hit of 0.001. We used as database the files of Regulatory Network Interactions (TF-gene interactions and Sigma – gene interactions interaction from the Regulon database (http://regulondb.ccg.unam.mx/). Using a Perl program, we obtained the TF’s and sigma factors that regulated each differentially expressed gene, using the bnumbers that we obtained by the Retrieve/ID mapping tool of Uniprot. Those results were visualized by Cytoscape [25].

### RT-qPCR analysis

To confirm RNA-seq results, some genes were selected for RT-qPCR analysis. Cultures were performed and sampled under the same conditions described for the RNA-seq experiments. Synthesis of cDNA and RT-qPCR conditions were followed as previously reported [26]. The primers employed in these experiments are listed in Additional file 1: Table S1.

### Generation of a pUA66 derivative with a *gfpmut2 *fusion to the promoter region of *aaeX*

Primers FaaeX (CGGGATCCCAAACACCACGATAACGG) and Raaex (CCGCTCGAGCTGATGGACGAAACGCTCA) were employed to amplify the *aaeR* promoter region. The PCR product was digested with enzymes BamHI and XhoI and cloned in plasmid pUA66. The resulting construction was verified by sequencing the cloned region.

## Results and discussion

### Growth kinetics of *Escherichia coli* W3110 in the presence of p-CA

**Fig. 1:**
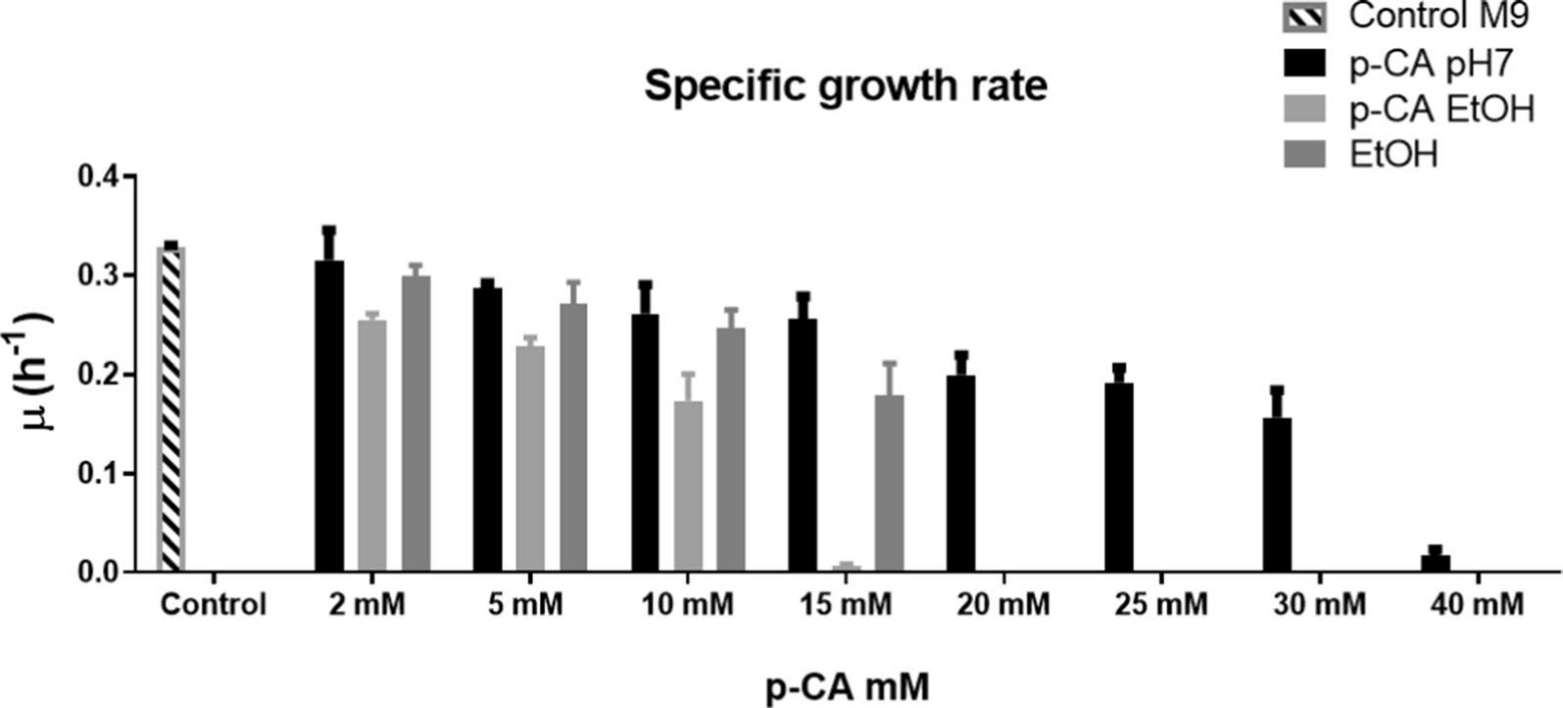
Specific growth rates of *E. coli* W3110 in the presence of p-CA. The p-CA was dissolved either in water adjusting pH to 7 or in absolute ethanol and M9 salts were added. As a control, cultures were grown in M9 medium. Cultures were performed in microtiter plates

To determine the effect of p-CA on the growth of *E. coli* strain W3110, cultures were performed in microtiter plates. Since p-CA displays low solubility in water, two different solutions were tested, one in water and another in ethanol. Figure 1 shows the specific growth rates (µ) observed from cultures in microtiter plates. It is evident that p-CA exerts a higher toxic effect when it is dissolved in ethanol when compared to its solution in water. These results show that p-CA is more toxic in its conjugated form. To perform global transcriptome analyses for studying the response to toxic compounds it is convenient that the concentration employed does not cause a reduction in µ larger than 28–33%, as this minimizes the possibility of detecting a general response to the reduced growth rate [27]. In these experiments, we observed a µ reduction with p-CA in ethanol at concentrations of 2, 5, and 10 mM corresponding to 20, 22 and 35%, respectively. Therefore, for the following studies, we decided to employ p-CA dissolved in ethanol at concentrations of 1.5, 3, and 5 mM.to avoid a severe reduction in growth rate.

### Dynamic transcriptional response of selected genes to p-CA

Experimental studies have identified genes that respond to p-CA and other aromatic acids in *E. coli* and *L. plantarum* [15, 20, 28]. Several of these genes were selected to determine the dynamic transcriptional response to p-CA. Genes related to stress functions were identified in *L. plantarum* growing in the presence of p-CA. From that group, we selected the following orthologous genes in *E. coli*: *clpB*, *clpP*, *dnaK* and *htpG*. Genes *clpB*, *dnaK* and *htpG* encode chaperone proteins and *clpP* a serine protease [29]. From a transcriptome study with *E. coli* in the presence of ferulic acid, we selected *marRAB*, the multiple antibiotic resistance operon, and gene *inaA*, encoding a pH-inducible putative lipopolysaccharide kinase involved in stress response [28]. Operon *aaeXAB* is the only group of genes known to be induced directly by p-CA in *E. coli* [15, 16]. This transcriptional unit, as well as its regulator *aaeR* were also included in this analysis.

For this part of the study, we utilized a library of promoter regions transcriptionally fused to *gfpmut2* in a low-copy plasmid [23]. The *E. coli* strain W3110 was transformed with plasmids containing the promoter fusion to *gfpmut2* for each of the genes mentioned above. This library did not contain the fusion of the promoter region of gene *aaeX* to *gfpmut2*. Therefore, this construct was generated by PCR amplifying and cloning in reverse the *aaeR* upstream region, as it also contains the divergent *aaeX* promoter. Strains containing plasmids with the transcriptional fusions were grown in microtiter plates where cell concentration and fluorescence were monitored. Two timeframes were defined for this part of the study, the long-term was extended up to 25 h whereas the short-term included up to 120 min after the addition of p-CA.

**Fig. 2:**
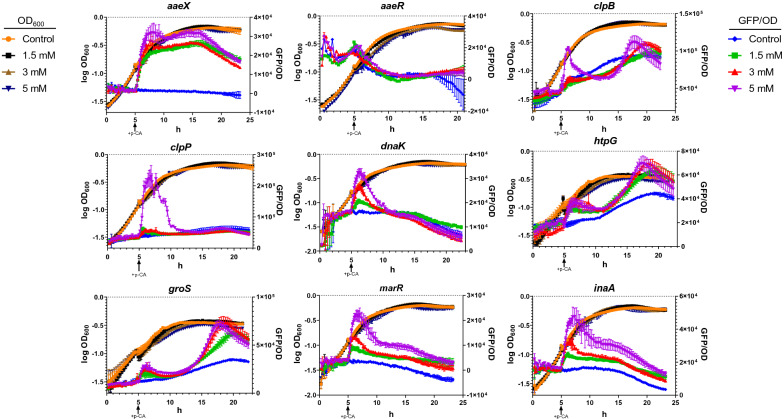
Long-term dynamic transcriptional response of selected genes and growth rates of *E. coli* W3110 in the presence of p-CA in microtiter cultures. The p-CA was dissolved in absolute ethanol at concentrations corresponding to 0- (control),1.5-, 3-, and 5-mM

The long-term responses of these genes to the presence of p-CA are shown in Fig. 2. In the case of gene *aaeX*, upon addition of p-CA induction was observed and this state was maintained during the stationary phase. The plasmid overexpression of operon *aaeXAB* has been reported to result in a two-fold increase in resistance to p-CA [10]. However, it has not been determined if this modification improves p-CA production capacity. In contrast, the expression level of gene *aaeR* remained relatively constant. Whereas in the case of genes *clpB*, *clpP*, *dnaK*, *htpG*, *groS*, *marR* and *inaA*, transient induction was observed with a peak upon addition of p-CA during the exponential growth phase and a subsequent reduction in expression level. In the case of genes *clpP*, *dnaK*, *marR* and *inaA* a single induction peak is observed immediately after addition of the aromatic acid. A different response is observed for genes *clpB*, *htpG* and *groS*, consisting of a first induction peak, and about 10 h later, a second higher-level induction peak. These patters provide useful information in the context of defining strategies for strain modification with the aim of increasing resistance to p-CA. The production processes for p-CA and derived products usually consist of cultures lasting several hours and include the stationary phase. The dynamic response data indicates that transcript levels of genes *clpP*, *dnaK*, and *inaA* increase and then sharply decrease in a few hours. It remains to be determined what would be the effect of maintaining a high-level expression of these genes by employing synthetic promoters, on the resistance to the toxic effect of p-CA and the production capacity for this aromatic acid.

**Fig. 3:**
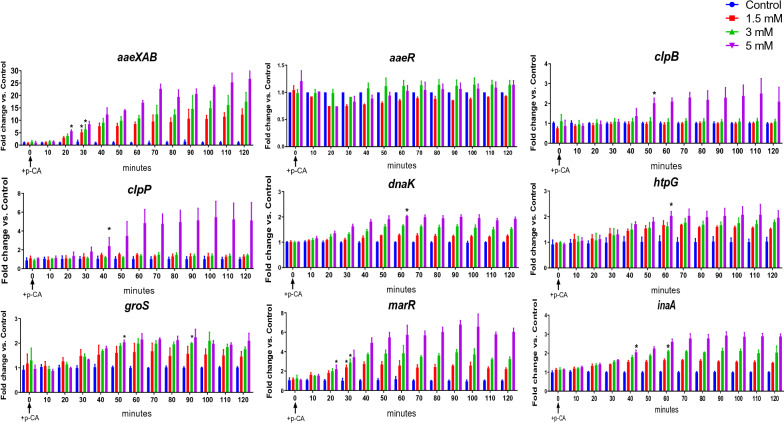
Short-term dynamic transcriptional response of selected genes to p-CA. Asterisks over bars indicate time and p-CA concentration that yields a twofold increase in specific fluorescence value. The p-CA was dissolved in absolute ethanol at concentrations corresponding to 0- (control),1.5-, 3-, and 5-mM

In the short-term experiments, we defined a positive response to p-CA when the fluorescence level was at least twice the value observed in the control cultures where only ethanol was added (Fig. 3). A *t*-test using the Holm-Sidak method was performed to determine significant differences at each time versus control. Induction of operon *aaeXAB* was detected 20 min after the addition of p-CA 5 mM and 30 min with p-CA 1.5 and 3 mM. The presence of p-CA did not influence the transcriptional activity of *aaeR*. For genes *clpB* and *clpP* fluorescence was detected only in cultures with p-CA 5 mM with induction observed after 50 and 40 min, respectively. In cultures with a strain having the *dnaK* transcriptional fusion to *gfpmut2*, fluorescence was observed 60 min after p-CA addition with 5 mM p-CA. Genes *htpG* and *groS* were found to be induced by 5 mM p-CA at 60 and 50 min, respectively. The protein products encoded by these genes are involved as part of cellular response to protein misfolding. These results strongly suggest that p-CA causes protein damage upon entry into the cytoplasm.

Induction of genes *marR* and *inaA* was observed at 20 and 40 min, respectively, after p-CA addition at all tested concentrations (Fig. 3). Gene *marR* belongs to the *marRAB* operon that is involved in multidrug resistance in *E. coli* by regulating efflux pump and porin expression. It is also known that MarA upregulates genes involved in lipid trafficking and DNA repair [30]. The gene *marR* encodes a repressor of the *marRAB* operon, while *marA* encodes an activator protein that autoactivates its operon. Operon *marRAB* is induced by the presence of several antibiotics as well as compounds with a phenolic ring [31]. Gene *inaA* is regulated by *marRAB* and *soxRS* [32]. Thus, *inaA* can be induced by the presence of compounds that induce *marRAB* and by the presence of superoxide stress via *soxRS*.

### Global transcriptional response of *E. coli* W3110 to p-CA

The results shown above indicated that several genes in *E. coli* respond to the presence of p-CA. To determine the global transcriptional response to p-CA, we performed RNA-seq analysis. Based on previous experiments, shake-flask cultures with *E. coli* W3110 were performed in the presence of 5 mM p-CA dissolved in ethanol that was added when an OD_600_ of 1.0 was reached, corresponding to the mid-exponential phase. Samples for RNA purification were taken at 20 and 60 min after the addition of p-CA. As a control, samples for RNA purification were taken from another culture without p-CA when it reached an OD_600_ of 1.0. Analysis of the RNA-seq data showed that 338 and 77 genes displayed a change in their expression level of at least twofold (*p* ˂ 0.05) after 20 and 60 min of addition of p-CA, respectively (Additional file 2: Table S2). The transcript level of some of the genes showing differential expression from the RNA-seq data and additional genes were confirmed by performing RT-qPCR analysis (Additional file 3: Table S3). An agreement regarding the transcriptional response was observed when comparing results with both methods.

**Fig. 4:**
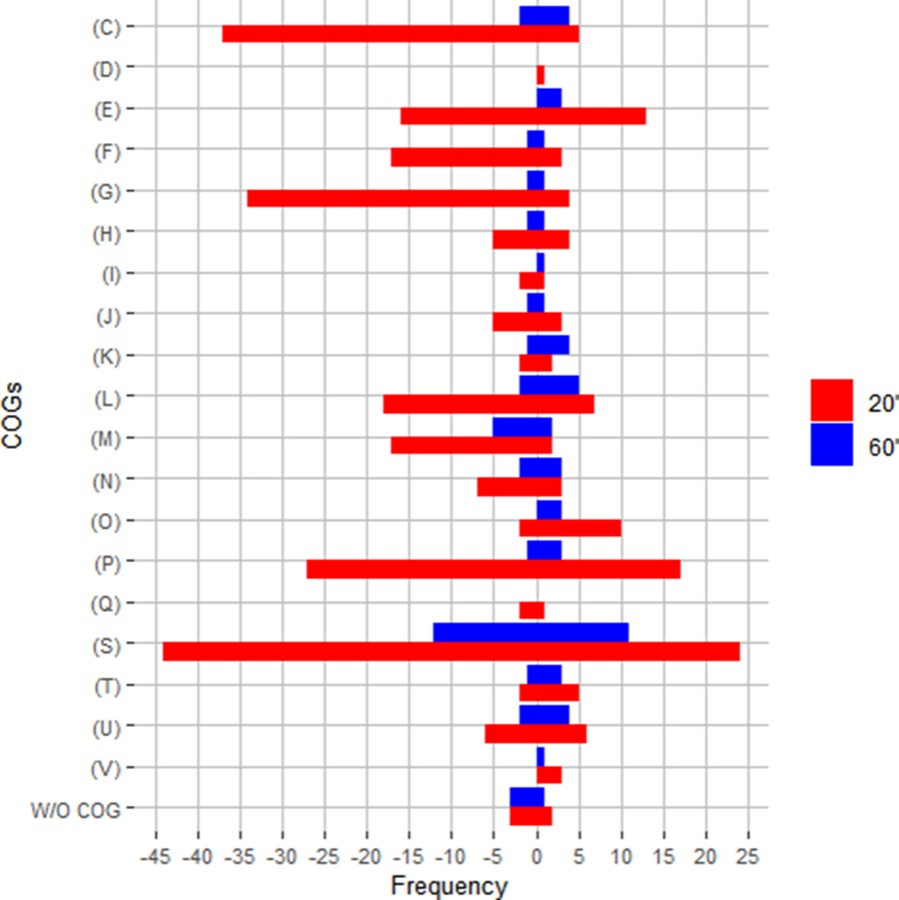
Differentially expressed genes arranged by Cluster of Orthologous Groups. Red and blue color bars indicate the number of genes responding 20 and 60 min after p-CA addition to the culture medium. Energy production and conversion (C), Cell cycle control and mitosis (D), Amino acid metabolism and transport (E), Nucleotide metabolism and transport (F), Carbohydrate metabolism and transport (G), Coenzyme metabolism (H), Lipid metabolism (I), Translation (J), Transcription (K), Replication, recombination and repair (L), Cell wall/membrane/envelop biogenesis (M), Cell motility (N), Post-translational modification, protein turnover, chaperone functions (O), Inorganic ion transport and metabolism (P), Secondary structure (Q), Signal transduction (T), Intracellular trafficking and secretion (U), Defense mechanisms (V)

Figure 4 shows the number of genes that were upregulated or downregulated in the presence of p-CA, grouped by functional category. These data provide a global view of the cell functions responding to the added stimulus. The transcript level of genes from several COG terms was found to change in the presence of p-CA. Some functional categories included genes displaying both increased and decreased transcript levels. However, in some cases, all the genes of a particular category displayed the same transcriptional response. A general trend towards reduction in transcript levels 20 min after the addition of p-CA to the culture medium was observed for the following categories: Energy production and conversion (C), nucleotide metabolism and transport (F), carbohydrate metabolism and transport (G), replication, recombination, and repair (L), cell wall/membrane/envelop biogenesis (M), cell motility (N) and genes that do not belong to a specific COG. Only two categories displayed an overall increased transcript response: post-translational modification, protein turnover, chaperone functions (O) and defense mechanisms (V). The response 60 min after the addition of p-CA followed the same general trend observed at 20 min but the absolute values differ. Therefore, the following detailed analysis was focused on the transcriptome data obtained 20 min after the addition of p-CA.

### Energy production, carbohydrate and nucleotide metabolism and transport

A general trend towards reduced transcript levels was observed for genes involved in energy production. This includes genes from TCA and aerobic respiration (*sdhABD*), anaerobic respiration (*nrfAD*, *hybAO* and *napAH*) and *atpF*, encoding the b subunit of the ATP synthase Fo complex. The downregulation of genes encoding proteins involved in carbohydrate transport was detected for D-galactose/methyl-galactoside (*mglABC*), mannitol (*cmtB*), rhamnose/lyxose (*rhaT*), maltose (*malK*) and trehalose (*treBC*). In addition, a lower transcript level was detected for genes encoding functions related to catabolism of sugars such as D-ribose (*mhpF*), D-allose (*alsE*), D-gulosides (*ycjQ*), galactose (*galK*), L-arabinose (*araBD*), L-xylose (*lyxK*, *sgbU*, *araD*), L-rhamnose (*rhaD*), lactose (*lacZ*) and N-Acetylneuraminate and N-Acetylmannosamine (*nanE*). Lower transcript levels were detected for purine nucleotide degradation (*psuk*) and pyrimidine nucleotide degradation (*mhpF* and *rihB*). It should be noted that none of these carbohydrates or nucleotides are present in the culture medium.

Exposure to p-CA resulted in the induction of the *aaeXAB* operon, identified as a tripartite export system for aromatic carboxylic acids, associated with p-hydroxybenzoic acid and p-CA export [16], being the main exporter of p-CA. The protein AaeR is the only known transcriptional factor in *E. coli* that responds directly to p-CA. As an early response to the presence of this aromatic acid, the operon *aaeXAB* is induced, thus activating a function that actively secretes the toxic compound. In addition to their role in carboxylic acid efflux, genes *aaeXAB* and *aaeR* have been involved in other cellular functions and growth conditions. Transcriptome analyses comparing planktonic with biofilm growth of urinary tract infection *E. coli* strains 83,972 and VR50 showed upregulation of genes from operon *aaeXAB*. When compared to a wild-type strain, a mutant with a deleted *aaeX* gene displayed a 33% reduction in biofilm formation. It is speculated that this efflux pump and others might be important for eliminating toxic intracellular compounds when cells are growing in a biofilm [32–34]. In another study, it was determined that in the absence of its effector pHBA, *aaeR* binds to several genes related to biofilm formation. In the absence of gene *aaeR*, biofilm formation was observed, suggesting a repression role of AaeR [18]. In addition, the protein encoded by *aaeR* is considered a virulence regulator in enterohemorrhagic and enteropathogenic *E. coli* strains. AaeR is activated by quorum sensing, and it participates in the activation of the expression of the locus of enterocyte effacement [17]. These data show that genes *aaeXAB* and *aaeR* have important roles in the physiology of *E. coli* even in the absence of p-CA or the effector pHBA. Therefore, the regulation of these genes is complex and could involve the integration of diverse internal and external signals.

A higher transcript level was detected for several genes encoding functions related to cell wall components synthesis. Gene *mtgA* encodes the enzyme MtgA, involved in the polymerization of lipid II molecules to form the glycan strands of peptidoglycan. The gene *ispE* is essential and encodes an enzyme that participates in the methylerythritol phosphate pathway I, leading to the synthesis of peptidoglycan [34]. This response is consistent with membrane damage, an occurrence in the presence of aromatic compounds in the medium [35–37]. The gene *lapB* was found upregulated in the presence of p-CA. This gene is known to be induced under heat shock conditions and it encodes an enzyme involved in the assembly of lipopolysaccharide (LPS) [38]. The higher transcript level of *lapB* might be a response to compensate membrane damage by p-CA. Genes related to cell cycle and division proteins were upregulated, including *tolA*, *tolR* and *zapA*. Proteins TolA, TolR and TolQ form a plasma membrane complex that is involved in maintaining outer membrane stability and participate in the cell division process.

The observed gene expression profile related to the active export of p-CA as well as cell wall and membrane repair and biogenesis shows the complex cellular response to contend with this toxic aromatic acid. Concomitant with mechanisms related to cell protection is the reduction in the intracellular concentration of p-CA resulting from the activity of the AaeXAB export system. This is complemented with a reduction in the transcript levels of genes encoding outer membrane and plasma membrane transport proteins, which could have the effect of decreasing permeability, therefore, reducing the rate of entry of p-CA into the cell. In addition, the upregulation of functions related to cell wall and membrane synthesis and repair is consistent with the known damage to these cell structures caused by several aromatic compounds. The lower expression levels of genes encoding proteins related to energy production can be explained considering the 25% reduction in growth rate caused by the presence of p-CA. The downregulation of transport and catabolic functions for substrates that are not present in the culture medium can be interpreted as an energy conservation strategy.

### Amino acid biosynthesis and transport

An increase in transcript level for several genes involved in amino acid biosynthesis was detected, including the pathways for the synthesis of alanine (*ilvE*), cysteine (*cysM*), isoleucine (*ilvADE*), leucine (*ilvE*), lysine (*lysA*), tryptophan (*trpAB*) and valine (*ilvDE*). This pattern suggests intracellular limitation of these amino acids. The increased expression level of gene *mtr* encoding a transporter for tryptophan supports a cellular state of tryptophan limitation. These data suggests that p-CA causes a partial depletion of some amino acids. This effect of p-CA could be explained considering that this compound disrupts membrane structure, thus causing the leakage of some intracellular compounds. This idea will be further discussed below. It has been reported that p-CA increases outer and inner membrane permeability as a damage mechanism [37].

### Signal transduction pathways

Genes *phoB* and *phoR* encode proteins that sense phosphate and regulate genes involved in phosphorus uptake and metabolism (Pho regulon). These two genes as well as members of the Pho regulon were found upregulated (*prpR*, *ompF*, *ugpA*, *ugpE*, *phoU*, *pstsA*, *pstsB*, *pstsC*, *pstsS*, *phnC*, *phnF*, *phnG*, *phnH*, *phnI*, *phnJ*, *phnM*, *phnN*). The observed response is consistent with the sensing of a phosphate limiting condition. This includes the higher transcript level of genes *pstABCS*, encoding periplasmic and membrane-bound components of the phosphate ABC transporter. The amount of phosphate is not limiting in the culture medium. Therefore, it can be speculated that this condition is caused by an alteration in the cell membrane, leading to leakage of cytoplasmic components, including molecules containing phosphate.

### Heat shock response

Genes belonging to the RpoH regulon were upregulated, including *clpB*, *dnaJ*, *dnaK*, *groL*, *groS*, *ibpA*, *ibpB*, *lapB*, *lon*, *yafE* and *zntR* [38]. The overexpression of chaperone and protease genes reflects oxidative stress and unfolded proteins response. Gene *spy* does not belong to the RpoH regulon but was found upregulated. It encodes an ATP-independent periplasmic chaperone that counteracts protein aggregation and promotes protein refolding [39]. The upregulation of these genes supports that p-CA causes oxidative damage to proteins, and their subsequent misfolding and aggregation.

### Multiple antibiotic resistance

Genes *marA* and *marR* were upregulated after 20 min of exposure to p-CA and a further increase in transcript level was observed at 60 min (Additional file 2: Table S2). Both genes are part of an operon including *marB*. MarA and MarB are transcriptional regulators whereas MarB is a periplasmic protein involved in multiple antibiotic resistance [40]. The gene *marR* encodes a repressor of the *marRAB* operon, while *marA* encodes an activator protein that autoactivates its operon. The presence of p-CA caused a positive response of the following genes from the MarA regulon: *acrA*, *acrB*, *tolC*, *lacZ*, *nfsA*, *rimK*, *fumC* and *ina.* Genes *acrA*, *acrB* and *tolC* encode proteins that constitute the AcrAB-TolC multidrug efflux pump. The upregulation of these three genes was detected in the RT-qPCR experiments (Additional file 3: Table S3). The AcrAB-TolC system exports many toxic compounds including organic solvents, dyes, antibiotics, and detergents [41]. Gene *inaA* is regulated by *marRAB* and *soxRS* [30]. Thus, *inaA* can be induced by the presence of compounds that induce *marRAB* and by the presence of superoxide stress via *soxRS*. Operon *marRAB* is induced by the presence of several antibiotics as well as compounds with a phenolic ring [31, 41–43]. These data indicate that p-CA can induce the MarA regulon, thus providing a response that can potentially lead to another route of export of this aromatic by the AcrAB-TolC pump.

### Thiol-specific oxidative damage and acid stress

The presence of p-CA elicited a thiol-specific oxidative damage response in *L. plantarum* [20]. In the case of *E. coli*, we observed a similar response characterized by the overexpression of genes related to sulfur amino acids biosynthesis, sulfate metabolism and assimilation, and thiol-specific oxidation. Also found induced were genes encoding proteins involved in glutathione (GSH) synthesis (*gshB)*, GSH import (*gsiA* and *gsiB)* and the GSH-dependent formaldehyde dehydrogenase (GS-FDH) (*frmA)*. The role of GS-FDH is related to the metabolism and detoxification of formaldehyde. Gene *frmA* is part of operon *frmRAB* that includes gene *frmB* encoding S-(hydroxymethyl)glutathione dehydrogenase and *fmrR* encoding the transcriptional repressor FmrR. This transcriptional regulator is known to bind formaldehyde, an event that causes induction of the *frmRAB* operon. Formaldehyde can be generated endogenously by several metabolic reactions, or it can enter the cell from an exogenous source [44]. It is not known that p-CA can lead directly to the generation of formaldehyde. Therefore, it is assumed that a metabolic imbalance caused by p-CA leads to formaldehyde accumulation that in turn induces expression of the *frmRAB* operon. Methylglyoxal (MG) is a toxic electrophile; it is a byproduct of glycolysis and is considered one of the main sources of endogenous formaldehyde in bacteria [45]. Formaldehyde is generated from MG during the Strecker degradation of glycine [46]. To contend with this issue, bacteria have pathways that participate in MG detoxification. One of them involves the transformation of MG to pyruvate. Transcriptome data showed that two genes from this pathway were downregulated: *gloA* and *lldD* encoding glyoxalase I and L-lactate dehydrogenase, respectively. Thus, it can be speculated that downregulation of *gloA* and *lldD* could cause the accumulation of MG, leading to its transformation to formaldehyde. It remains to be determined how p-CA causes a reduction in the transcript level of these two genes.

Gene *yhcN* displayed upregulation in response to p-CA. Protein YhcN is uncharacterized, however, it has been associated with acid stress, biofilm formation, and *cys*-dichloroethylene degradation in *E. coli* [15]. This gene was found to be upregulated as a response to cytoplasmic acid stress by the addition of the permeant weak acid benzoate [47, 48]. This gene is considered a reporter for cytoplasmic acid stress, indicating that p-CA causes this type of stress upon entry into the bacterial cytoplasm.

### Mutant characterization to determine contribution of specific genes to p-CA resistance

**Fig. 5:**
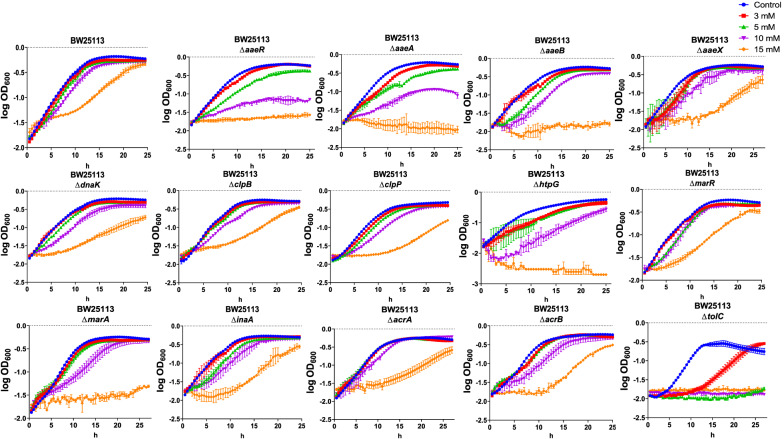
Growth of *E. coli* BW25113 and derived mutants in genes *aaeR*, *aaeA*, *aaeB*, *aaeX*, *dnaK*, *clpB*, *clpP*, *htpG*, *marR*, *marA*, *inaA*, *acrA*, *acrB* or *tolC*. Cultures were performed in microtiter plates containing medium with 0- (control), 3-, 5-, 10- and 15-mM p-CA dissolved in ethanol

Gene expression data from RNA-seq and RT-qPCR showed upregulation of genes encoding components of efflux systems and regulatory proteins that could have a role in providing resistance to p-CA toxicity. To determine the contribution of each of these genes, the growth of wild type BW25113 as well as mutants lacking functional genes *aaeR*, *aaeA*, *aaeB*, *aaeX*, *dnaK*, *clpB*, *clpP*, *htpG*, *marR*, *marA*, *inaA*, *acrA*, *acrB* or *tolC* was compared in the presence of 0, 3, 5, 10 and 15 mM p-CA (Fig. 5 and Additional file 4: Table S4). The growth rate of wild type strain BW25113 was reduced only in the presence of 15 mM p-CA. When compared to BW25113, the Δ*aaeR* and the Δ*aaeA* mutants displayed reduced resistance to all tested concentrations of p-CA. A similar response but only at the two higher p-CA concentrations was observed in strain Δ*aaeB*. The elimination of *aaeX* caused a minor change in growth rate at 15 mM p-CA when compared to the wild type strain. These results provide novel quantitative data and confirm published reports showing that proteins AaeA and AaeB function as components of a p-CA efflux pump [10, 16]. The function of AaeX is unknown, and our results indicate that this protein does not have an important role in p-CA efflux. Protein AaeR is a positive transcription factor for the *aaeXAB* operon [16]. The data from this study indicates that elimination of *aaeR* causes a severe disruption of p-CA efflux function.

The response observed in strains lacking genes *dnaK*, *clpB* or *clpP* showed reduced resistance to p-CA at the two higher concentrations. In the case of the Δ*htpG* mutant, it displayed lower growth at all tested p-CA concentrations. This is the first reported evidence showing the importance of chaperone, disaggregase and protease activities on resistance to p-CA and is indicative of protein damage upon exposure to this aromatic acid. These results suggest that overexpression of these genes could lead to increased resistance to p-CA and improved production strain performance. Gene *htpG* encodes Hsp90, an ATP-dependent chaperone. In a study to determine the regions of Hsp90 that are important for client binding, gene *htpG* was overexpressed in *E. coli*. It was determined that high level expression of *htpG* from an arabinose-inducible promoter resulted in a decrease in colony-forming units. These cells were also found to be filamentous [49]. These data indicate that an strategy based on *htpG* expression for improving p-CA resistance in *E. coli* will require careful promoter selection to avoid an excessive and detrimental level of Hsp90.

The inactivation of *inaA* caused a reduction in resistance to p-CA mainly when it is present at a 15 mM concentration, and to a lesser extent at lower concentrations. Expression of gene *inaA* is dependent on the multiple antibiotic resistance and superoxide stress response systems, as well as the DNA-binding transcriptional dual regulator Rob. However, the role of InaA in these responses is not known. The results obtained here show that InaA contributes to the resistance to p-CA by an unknown mechanism.

The growth rate of the Δ*tolC* mutant was highly reduced for all tested p-CA concentrations, as it has been shown previously [10]. Protein TolC is the common outer membrane channel of several efflux systems that eliminate toxic compounds from the cytoplasm and periplasm. TolC participates in the efflux of p-CA and contributes with AaeAB to resistance to this toxic compound. Protein TolC can form a complex with the AcrAB efflux pump. Genes *acrA* and *acrB* showed induction in the presence of p-CA in the RT-qPCR experiments. In the presence of 15 mM p-CA, a reduction in growth was observed for the Δ*acrB* strain, and no change for Δ*acrA*. These results indicate that AcrAB in complex with TolC, plays a minor role in p-CA efflux [10]. It is evident that Δ*tolC* causes a reduction in growth rate of a larger magnitude than Δ*acrB.* This result is explained considering that TolC can form complexes with several other efflux pumps different from AcrAB, such as AcrEF, EmrAB, EmrKY, MacAB, MdtABC and MdtEF [50]. Therefore, one or several of these complexes with TolC are participating in the efflux of p-CA. A reduction in growth rate was detected for mutant Δ*marA* at 10- and 15-mM p-CA, whereas no effect was observed for the Δ*marR* strain (Fig. 5). Protein MarA is an activator protein involved in induction of several genes including *acrA*, *acrB* and *tolC* [31]. These data show the important role of MarA on reducing the negative impact of p-CA on the cell.

In this report, the transcriptome response of *E. coli* to p-CA was characterized. This toxic compound impacts several functions and causes a response involving export proteins. The identification of genes that contribute to p-CA resistance in *E. coli* such as those involved in small molecule efflux and multiple antibiotic resistance, provide targets for improving p-CA production strains. The further experimental analysis of the genes displaying differential expression in the presence of p-CA will help in understanding their role in the physiology of *E. coli* growing in a toxic environment and might also yield further targets for production strain improvement.

## Supplementary Information

**Additional file 1: Table S1.** Primers sequence of genes for RT-qPCR.

**Additional file 2: Table S2.** Transcript levels of differentially expressed genes after 20 and 60 minutes of exposure to p-CA.

**Additional file 3: Table S3.** RT-qPCR analysis of selected genes.

**Additional file 4: Table S4.** Specific growth rates of mutants in the presence of 0 (control), 3, 5, 10 and 15 mM of p-CA.

## Data Availability

The datasets used and/or analyzed during the current study are available from the corresponding authors on reasonable request.

## Abbreviations

PAL: Phenylalanine ammonia lyase
TAL: Tyrosine ammonia lyase
p-CA: *p*-Coumaric acid
C4H: Cinnamate 4-hydroxylase
MIC: Minimal inhibitory concentration
COG: Cluster of orthologous groups
µ: Specific growth rate
LPS: Lipopolysaccharide
H_2_O_2_: Hydrogen peroxide
GSH: Glutathione
GS-FDH: GSH-dependent formaldehyde dehydrogenase
