# Supplementary material for: Global transcriptomic response of Escherichia coli to p-coumaric acid
Source: Microb Cell Fact. 2022 Jul 20;21:148. doi: 10.1186/s12934-022-01874-6 (PMC9301823; doi:10.1186/s12934-022-01874-6)
Supplement: Supplementary file 1 — Additional file 1: Table S1. Primers sequence of genes for RT-qPCR. [file 12934_2022_1874_MOESM1_ESM.docx]

Table S1. Primers sequence of genes for RT-qPCR.

| Primer name | Sequence |
| --- | --- |
| aaeXF | CCGTTATCGTGGTGTTTGGG |
| aaeXR | TGTTGGCACAAGTACCCGG |
| aaeAF | AATGCCTGGGTCTATTACACCG |
| aaeAR | TGGGTAATGAGTCCAGAAACGTC |
| aaeBF | AGACCATGTGATATCTGCCCG |
| aaeBR | GCCAGATGCGTAGCTTTTCCT |
| aaeRF | CCGCCAGACAGCTACAGATG |
| aaeRR | GCTGCGTGTGCTACGGTTTAA |
| acrAF | CAGAGGGTTTACGCCTCTGG |
| acrAR | CATCTGCTGGCCACCTTGTT |
| acrBF | TGGGTGATGTTCAGTTGTTCG |
| acrBR | GGTAATGACATCAACCGGCG |
| tolCF | GCCTGAGCCTTTCTGGGTTC |
| tolCR | GGCAGACTTACGCAATTCCG |
| clpBF | GAACAAATGCGTGGAGGTGA |
| clpBR | TGTTCGGCTCGTTCGGTAA |
| clpPF | CTGATTGTGGCGCAGATGC |
| clpPR | GACATCCCGGCAGTGATCAC |
| clpXF | GATGTTCCGTTCACCATGGC |
| clpXR | TCGCATTTCTGCAACAGCTT |
| dnaJF | AACAGCACCCGATTTTCGAG |
| dnaJR | GGTCGGTACTTCGATTTCGC |
| groLF | GTGGTGGTGTTGCGCTGAT |
| groLR | CATTGCACGCAGTGCAACTT |
| marRF | CCTGGTCTGTAAAGGCTGGG |
| marRR | ATTGTTCACATATTGCCGCG |
| marAF | AAGTGTCAGAGCGTTCGGGT |
| marAR | CATCTTACGGCTGCGGATGT |
| marBF | TTCATCCGCAATAGCAGCTG |
| marBR | CAACCACGACATTGGCACAA |
| inaAF | TCCGTACGTTATCCGTTCGG |
| inaAR | CCAAAAACGATCTTCGGCAC |
