# Supplementary material for: Global transcriptomic response of Escherichia coli to p-coumaric acid
Source: Microb Cell Fact. 2022 Jul 20;21:148. doi: 10.1186/s12934-022-01874-6 (PMC9301823; doi:10.1186/s12934-022-01874-6)
Supplement: Supplementary file 3 — Additional file 3: Table S3. RT-qPCR analysis of selected genes. [file 12934_2022_1874_MOESM3_ESM.docx]

Table S3. RT-qPCR analysis of selected genes.

|  | **2^-ddCt^** | | |
| --- | --- | --- | --- |
|  | **Control** | **pHCA 5 mM 20´** | **pHCA 5 mM 60´** |
| **Gen** | **Aromatic acid efflux system AaeXAB** | | |
| ***aaeX*** | **1** +/- 0.08 | **205.8** +/- 27.6 | **52.6** +/- 7 |
| ***aaeA*** | **0.9** +/- 0.12 | **107** +/- 8.4 | **20.9** +/- 4 |
| ***aaeB*** | **0.9** +/- 0.1 | **470** +/- 70.6 | **63.3** +/- 17.3 |
| ***aaeR*** | **1** +/- 0.03 | **1.7** +/- 0.2 | **0.8** +/- 0.12 |
|  | **Chaperones and proteases** | | |
| ***clpB*** | **1.3** +/- 0.3 | **18.9** +/- 1.7 | **6.5** +/- 0.92 |
| ***clpP*** | **0.87** +/- 0.14 | **3.3** +/- 0.59 | **1.2** +/- 0.11 |
| ***clpX*** | **1** +/- 0.03 | **3.8** +/- 0.29 | **1.6** +/- 0.24 |
| ***dnaJ*** | **1.1** +/- 0.12 | **10.8** +/- 1.6 | **3.3** +/- 0.4 |
| ***groL*** | **1.1** +/- 0.27 | **12.7** +/- 1.95 | **5.6** +/- 0.33 |
|  | **Multridug resistance** | | |
| ***marR*** | **0.8** +/- 0.13 | **161.1** +/- 42.1 | **101.1** +/- 25.12 |
| ***marA*** | **1** +/- 0.05 | **200.7** +/- 11 | **43.5** +/- 5.8 |
| ***marB*** | **1** +/- 0.11 | **162.2** +/- 6.9 | **19.7** +/- 0.12 |
| ***inaA*** | **1.1** +/- 0.12 | **23** +/- 0.29 | **5.9** +/- 0.97 |
|  | **Multridug efflux system AcrAB-TolC complex** | | |
| ***acrA*** | **0.96** +/-0.02 | **2.9** +/- 0.32 | **1.5** +/-0.3 |
| ***acrB*** | **1** +/- 0.06 | **3.5** +/-0.33 | **1.3** +/-0.19 |
| ***tolC*** | **1** +/- 0.05 | **2.8** +/- 0.06 | **2** +/- 0.25 |
