# Supplementary material for: Global transcriptomic response of Escherichia coli to p-coumaric acid
Source: Microb Cell Fact. 2022 Jul 20;21:148. doi: 10.1186/s12934-022-01874-6 (PMC9301823; doi:10.1186/s12934-022-01874-6)
Supplement: Supplementary file 4 — Additional file 4: Table S4. Specific growth rates of mutants in the presence of 0 (control), 3, 5, 10 and 15 mM of p-CA. [file 12934_2022_1874_MOESM4_ESM.docx]

Table S4. Specific growth rates of mutants in the presence of 0 (control), 3, 5, 10 and 15 mM of p-CA.
